# Improvement of *In Vivo* Fluorescence Tools for Fast Monitoring of Freshwater Phytoplankton and Potentially Harmful Cyanobacteria

**DOI:** 10.3390/ijerph192114075

**Published:** 2022-10-28

**Authors:** Mara Simonazzi, Laura Pezzolesi, Franca Guerrini, Silvana Vanucci, Giancarlo Graziani, Ivo Vasumini, Andrea Pandolfi, Irene Servadei, Rossella Pistocchi

**Affiliations:** 1 Department of Biological, Geological and Environmental Sciences (BiGeA), University of Bologna, Via S’Alberto 163, 48123 Ravenna, Italy; 2 Interdepartmental Centre for Industrial Research in Renewable Resources, Environment, Sea and Energy (CIRI-FRAME), University of Bologna, Via S’Alberto 163, 48123 Ravenna, Italy; 3 Department of Chemical, Biological, Pharmaceutical and Environmental Sciences (ChiBioFarAm), University of Messina, Viale Ferdinando d’Alcontres 31, 98166 Messina, Italy; 4 Romagna Acque Società delle Fonti S.p.a., Piazza Orsi Mangelli 10, 47122 Forlì, Italy; 5 Fondazione Centro Ricerche Marine, Viale A. Vespucci, 2, 47042 Cesenatico, Italy

**Keywords:** chlorophyll *a*, harmful cyanobacteria, multi-wavelength spectrofluorometers, *in vivo* fluorescence, chlorophyll extraction, intercalibration studies

## Abstract

The use of multi-wavelength spectrofluorometers for the fast detection of algal taxa, based on chlorophyll *a* (Chl-*a*) emission spectra, has become a common practice in freshwater water management, although concerns about their accuracy have been raised. Here, inter-laboratory comparisons using monoalgal cultures have been performed to assess the reliability of different spectrofluorometer models, alongside Chl-*a* extraction methods. Higher Chl-*a* concentrations were obtained when using the spectrofluorometers than extraction methods, likely due to the poor extraction efficiencies of solvents, highlighting that traditional extraction methods could underestimate algal or cyanobacterial biomass. Spectrofluorometers correctly assigned species to the respective taxonomic group, with low and constant percent attribution errors (Chlorophyta and Euglenophyceae 6–8%, Cyanobacteria 0–3%, and Bacillariophyta 10–16%), suggesting that functioning limitations can be overcome by spectrofluorometer re-calibration with fresh cultures. The monitoring of a natural phytoplankton assemblage dominated by Chlorophyta and Cyanobacteria gave consistent results among spectrofluorometers and with microscopic observations, especially when cell biovolume rather than cell density was considered. In conclusion, multi-wavelength spectrofluorometers were confirmed as valid tools for freshwater monitoring, whereas a major focus on intercalibration procedures is encouraged to improve their reliability and broaden their use as fast monitoring tools to prevent environmental and public health issues related to the presence of harmful cyanobacteria.

## 1. Introduction

The occurrence of cyanobacterial blooms is an ever-increasing phenomenon in freshwater ecosystems and their frequency and severity has been forecast to further increase in the upcoming years due to eutrophication and climate changes [1]. The presence of cyanobacteria in water bodies intended for drinking purposes is potentially harmful, since several species have been reported to produce cyanotoxins, which may affect both animals and humans through the ingestion of contaminated water, skin contact, or aerosol inhalation [2]. Among the cyanobacterial toxins, the hepatotoxic microcystins are considered the most widespread in freshwater and comprise more than 250 known variants, which act as potent inhibitors of protein phosphatases. Their presence in water bodies has been linked to the death of several animals, and human fatalities due to liver failure have also occurred [1,3]. The ability to synthesize microcystins is shared by some freshwater cyanobacteria, including the widespread bloom-forming genera *Microcystis*, *Planktothrix*, and *Dolichospermum*. Other frequently detected cyanotoxins are the neurotoxic anatoxin-a and saxitoxins, both produced by various species belonging to the genera *Anabaena*, *Dolichospermum*, and *Aphanizomenon*; cylindrospermopsin, an alkaloid first isolated in *Cylindrospermopsis raciborskii*, is reported to be able to target multiple organs and inhibit protein synthesis in plants and animals [1]. The surveillance of cyanobacteria in water bodies is usually guaranteed through samplings, followed by the identification and cell counting of phytoplankton often associated with the estimation of biovolume. Although these approaches provide detailed information, they are time consuming and require highly qualified operators able to identify species with a certain degree of confidence [4]. Moreover, an increase in variability is expected as a result of sample transport and storage [5], as well as of possible cellular shrinkage due to preservation with Lugol’s iodine solution, hence inaccuracies in biovolume determination [6]. Chlorophyll *a* (Chl-*a*) is the universal pigment shared by all photosynthetic organisms and represents a useful parameter in the monitoring of their abundances in aquatic ecosystems, although it is not informative regarding phytoplankton community composition [7]. Chl-*a* is usually extracted using organic solvents (e.g., acetone, ethanol, methanol, and dimethyl sulfoxide), and subsequently determined spectrophotometrically [8] or through a chemotaxonomic approach via HPLC [9], a methodology not suitable for real-time measurements. Specific pigments such as phycocyanin (PC) and phycoerythrin (PE) could be analyzed to determine cyanobacteria presence; however, poor extraction efficiency may occur, leading to possible underestimation of the real concentrations [10]. The implementation of detection tools that rely on pigments’ fluorescence (i.e., *in vivo* fluorescence) can be considered as a useful supportive system in drinking water monitoring. Among commercially available sensors, multi-wavelength spectrofluorometers have been widely tested for monitoring cyanobacterial blooms in lakes and drinking water reservoirs [5,7,11,12,13] and, more recently, also for benthic communities [14,15]. These probes have a user-friendly approach and can give real-time information on phytoplankton community composition, providing the relative abundance of certain algal groups [16]. Algal group discrimination by *in vivo* fluorescence is based on the presence of diagnostic pigments that influence the emission spectrum of Chl-*a*, after pigments’ excitation with multiple wavelengths, resulting in a spectral “fingerprint” for each algal taxon considered [17]. Typically, four broad algal groups can be determined with such an approach: the “green” group (Chlorophyta and Euglenophyceae), Cyanobacteria (PC-rich), the “brown” group (Diatoms, Chrysophytes, and Dinoflagellates), and the “red” group (Cryptophyta and other PE-containing organisms); additionally, it would potentially be possible to calibrate these probes for other species-specific spectral “fingerprints” [13]. In general, good agreement between the data from spectrofluorometers and traditional methodologies was found, i.e., in terms of the Chl-*a* extracted, the cell counting, and the biovolume [5,7,11,12,18,19,20], whereas in some cases, weak relationships among the methods were reported [21,22]. The main limitations and interference sources include the high turbidity of water and a high number of cells, the presence of chromophoric dissolved organic matter (cDOM), cellular agglomeration and colonial or filamentous organisms, different morphology and dimension of cells, and variations in light, nutrients, and temperature [16,23]. It is known that the use of different solvents may lead to higher or lower extraction efficiencies; thus, variation in Chl-*a* concentrations is expected. Additionally, as these probes are designed to address various applications and needs, i.e., *in situ* real-time measures at a fixed depth or, alternatively, measurement of the whole water column profile, discrepancies in determinations due to their characteristics rather than to the methodology used should not be excluded, especially when comparing different sensors with each other.

The main objective of the study was to improve the fast monitoring and management of freshwaters, including those intended for drinking water, using multi-wavelength spectrofluorometers to prevent environmental and public health issues related to the presence of harmful cyanobacteria.

This was achieved using an intercalibration approach among seven distinct laboratories: (i) comparatively assessing the performance of seven multi-wavelength spectrofluorometers belonging to four different models (all provided by bbe Moldaenke, Germany) in detecting algae and cyanobacteria using monoalgal cultures; (ii) comparing the *in vivo* fluorescence results with the Chl-*a* values obtained with different extraction methods, i.e., 90% acetone, 90% ethanol, and dimethyl sulfoxide; and (iii) evaluating the reliability of the probes tested on a field sample.

## 2. Materials and Methods

### 2.1. Setup of Monoalgal Cultures 

A total of 12 freshwater phytoplankton strains belonging to distinct algal groups, i.e., the “green” group, Cyanobacteria, and the “brown” group, were employed in this study (Table 1). All strains were obtained from international culture collections except (i) *Desmodesmus communis*, that was isolated as described in Samorì et al. [24], and cf. *Dolichospermum* sp., cf. *Anabaena* sp., and cf. *Cyclotella* sp., that were isolated through manual pipetting under the microscope from surface water collected from Reno River (Ravenna, Emilia-Romagna, Italy) in July 2018, and morphologically identified at the genus level using an inverted light microscope at 320x magnification (ZEISS Axiovert 100). Algae from the “green group”, *Chlorella vulgaris* (both strains), and *D. communis* were grown in modified CHU 13 medium [25], while *Euglena gracilis* was cultured in Cramer–Myers medium [26]. All Cyanobacteria were grown in BG11 medium [27], except for cf. *Dolichospermum* sp. and cf. *Anabaena* sp. that were maintained in Jaworski medium [28]. *Stephanodiscus hantzschii*, *Fragilaria crotonensis*, and cf. *Cyclotella* sp. were grown in Diatom medium [29]. All cultures were maintained in 250 mL glass Erlenmeyer flasks at 20 ± 1 °C, a light intensity of 90–110 μmol photons m^−2^ s^−1^, and a light:dark photoperiod of 16:8 h.

### 2.2. Sample Preparation for Intercalibration Procedures

A total of 23 simultaneous tests for Chl-*a* determination were performed on monoalgal cultures by seven distinct laboratories, namely, Lab1–7, using either spectrofluorometers (Lab1–5) or extraction methods (Lab6 and Lab7). The monoalgal cultures were scaled up by Lab1 under the same conditions as described above (Section 2.1), to reach appropriate volumes for Chl-*a* intercalibration (5–10 L). Culture aliquots were collected during the exponential growth phase to avoid the degradation of photosynthetic pigments, which may take place during the stationary growth phase; the aliquots were put in 1 L polypropylene sampling bottles to provide samples to all of the other laboratories (Lab2–7), located in different parts of Italy. The bottles were kept at 4 °C in the dark until Chl-*a* measurement, which took place within 24 h from the samplings. Each sampling bottle was adapted to room temperature and gently mixed upside-down at least 30 times to avoid algal sedimentation, and then dilutions with distilled water (between 1:2 and 1:40) were performed to obtain Chl-*a* concentrations suitable for *in vivo* fluorescence analysis, i.e., 0–200 µg Chl-*a* L^−1^. All determinations were performed under ambient low light to avoid photoinhibition and fluorescence quenching, and thus Chl-*a* underestimation [30]. 

### 2.3. Determination of Chl-a Using In Vivo Fluorescence

Measurements of Chl-*a* were performed using seven bbe Moldaenke spectrofluorometers providing the simultaneous detection of four algal groups based on their specific fluorescence emission spectra, i.e., the “green” group, Cyanobacteria, the “brown” group, and Cryptophyta (bbe Moldaenke GmbH, Kiel, Germany). Four models of bbe spectrofluorometers were employed (Table S1): AlgaeLabAnalyser (ALA), FluoroProbe (FP), four AlgaeOnlineAnalysers (AOAs, i.e., AOA1, AOA2, AOA3, and AOA4, sharing all of the technical specifications), and AlgaeTorch (AT). ALA was a laboratory-based spectrofluorometer equipped with a 25 mL glass cuvette; FP was an *in vivo* portable probe for field applications and depth profile measurements; the four AOAs (AOA1–4) were *in situ* spectrofluorometers providing real-time Chl-*a* measurements; AT was a portable probe designed to only measure total and cyanobacterial Chl-*a* (see Supplementary Materials, Table S1). ALA, FP, and AOA were calibrated by the manufacturer for standard algal group differentiation and were specifically equipped with a dedicated channel for the detection of *Planktothrix rubescens*. All spectrofluorometers except AT were equipped with an additional UV LED (370 nm) to correct the measurements to the “yellow substances” (i.e., cDOM). During the tests, the dedicated channel for Cryptophyta detection was turned off for all probes, to adopt the standard setup used by the laboratories involved in the study for the routine analyses of field samples. The channel for *P. rubescens* detection was only activated for monospecific cultures of the cyanobacterium and for the field sample.

### 2.4. Determination of Chl-a Using Extraction Methods

In parallel to spectrofluorimetric analysis, Chl-*a* was determined spectrophotometrically (UV/VIS, JASCO V-650, Tokyo, Japan), using different procedures and solvents for the extraction, i.e., 90% acetone (ACT), 90% ethanol (EtOH), and dimethyl sulfoxide (DMSO). All measurements were performed in triplicate.

#### 2.4.1. Solvent 1: 90% Acetone

Aliquots of diluted algal cultures (50–100 mL) were filtered on nitrocellulose filters (Whatmann, nominal porosity 0.45 µm, Ø 47 mm) and transferred to test tubes where 10 mL of acetone (90% *v*/*v*) was added. The samples were vortexed until complete dissolution of the filter, and then dark-incubated at 4 °C for 20–24 h. After incubation, 5 mL of solvent was added to the samples, which were then vortexed and centrifuged at 2550× *g* for 10 min. For monoalgal samples, the absorbance of the extracts was measured at 630, 647, 664, and 750 nm and the concentration of Chl-*a* was calculated according to the specific algal groups and solvent equations proposed by Ritchie [8]. For the field sample, an additional reading at 691 nm was conducted and the Chl-*a* content was calculated according to the quadrichroic equation proposed by Ritchie [31].

#### 2.4.2. Solvent 2: 90% Ethanol

Determination using ethanol was performed according to the standard procedure ISO 10260:1992, variant A [32]. Briefly, aliquots of diluted samples (50–100 mL) were filtered on glass microfiber filters (Whatman GF/F, nominal porosity 0.7 µm, Ø 47 mm), cut into pieces, and transferred into test tubes. The Chl-*a* was extracted with 10 mL hot ethanol (90%, *v*/*v*) at 78–80 °C for 10 min, and then stored at room temperature in the dark overnight. After 20–24 h, the samples were centrifuged at 2550× *g* for 10 min and spectrophotometric readings of the extracts were performed at 629, 649, 665, 696, and 750 nm for the monoalgal samples, calculating the Chl-*a* concentration based on the algal group formulae proposed for ethanol by Ritchie [8]. For the field sample, the 629 nm wavelength was substituted with 632 nm and the Chl-*a* was quantified according to the quadrichroic equation [31].

#### 2.4.3. Solvent 3: Dimethyl Sulfoxide

The filtration of the diluted samples was performed as reported for ethanol without cutting the filters, and the Chl-*a* was extracted in 7 mL of dimethyl sulfoxide (DMSO) at 60–65 °C for 10 min. After cooling down the samples for 15 min in the dark at room temperature, the extracts were vortexed and centrifuged at 2550× *g* for 10 min. Absorbance readings were performed at 649, 665, and 750 nm and calculations were made as reported by Wellburn [33].

### 2.5. Performance Evaluation of the Probes

The relative variation of the Chl-*a* concentrations, as determined by each model of the probes, was evaluated in terms of coefficient of variation (CV%), compared to the general mean of the extraction methods per each monoalgal sample, and calculated as follows:

CV(%) = (sd(Chl-*a*_PROBE_)/mean(Chl-*a*_EXTR_))∙100 (1)

where:

sd(Chl-*a*_PROBE_) = standard deviation of the concentration of Chl-*a* (µg L^−1^) as measured by a model of the probes, i.e., ALA, FP or AOAs;

mean(Chl-*a*_EXTR_) = general mean of the concentration of total Chl-*a* (µg L^−1^) for each monoalgal sample as measured by all extraction methods, i.e., ACT, EtOH and DMSO.

The algal group assignment by spectrofluorometers was evaluated as percent error (Er%) in terms of attribution of the target algal group with respect to the total content of Chl-*a*, as follows:

Er(%) = (|Chl-*a*_GROUP_ − Chl-*a*_TOT_|/Chl-*a*_TOT_)∙100 (2)

where:

Chl-*a*_GROUP_ = concentration of Chl-*a* (µg L^−1^) of the target algal group (“green” group, Cyanobacteria, or “brown” group) for each sample;

Chl-*a*_TOT_ = concentration of total Chl-*a* (µg L^−1^) of each sample. 

The percent accuracy (Acc%) was consequently determined by subtracting the Er% to a perfect fit of 100%:

(3) Acc(%)=(100%−Er%)·100

### 2.6. Application of Probes to a Field Freshwater Sample

Spectrofluorometers and extraction methods were used for Chl-*a* determination on a field freshwater sample collected from Reno River (Ravenna, Italy) in September 2020. Subsamples were prepared in 1 L polypropylene bottles, delivered to the laboratories, and maintained as described for the monoalgal cultures prior to Chl-*a* analysis (Section 2.2). All determinations were simultaneously performed on undiluted samples by each laboratory within 24 h of the sampling. A sub-sample was fixed with Lugol’s iodine solution and qualitative and quantitative analyses of phytoplankton and Cyanobacteria were performed according to Utermöhl’s method [34] using the same microscope as described before (Section 2.1). For filamentous Cyanobacteria, counting was performed either in terms of cell L^−1^ or filaments L^−1^; the number of cells per filament was obtained by measuring the total length of filaments (µm) and dividing it by the average cell’s height (µm), as measured in a consistent number of individuals (*n* ≥ 30). The biovolume of each species was determined according to Napiórkowska-Krzebietke and Kobos [35], and expressed as mm^3^ L^−1^.

### 2.7. Data Analysis

The statistical analyses were performed on PAST version 4.09 [36]. The data homogeneity assumption was checked with Levene’s test (from medians) and was found to be not significant. The Kruskal–Wallis test was used for the univariate analysis of Chl-*a* concentrations among each method based on algal groups and significant comparisons were assessed with a Mann–Whitney pairwise post hoc test. Where appropriate, an analysis of variance (ANOVA) was used, followed by a post hoc Tukey’s HSD test. The comparison of the probes’ output against the cell counts and biovolumes of the field sample was assessed via a Student’s *t*-test.

## 3. Results

### 3.1. Evaluation of Different Solvents for Chl-a Extraction

**Figure 1:**
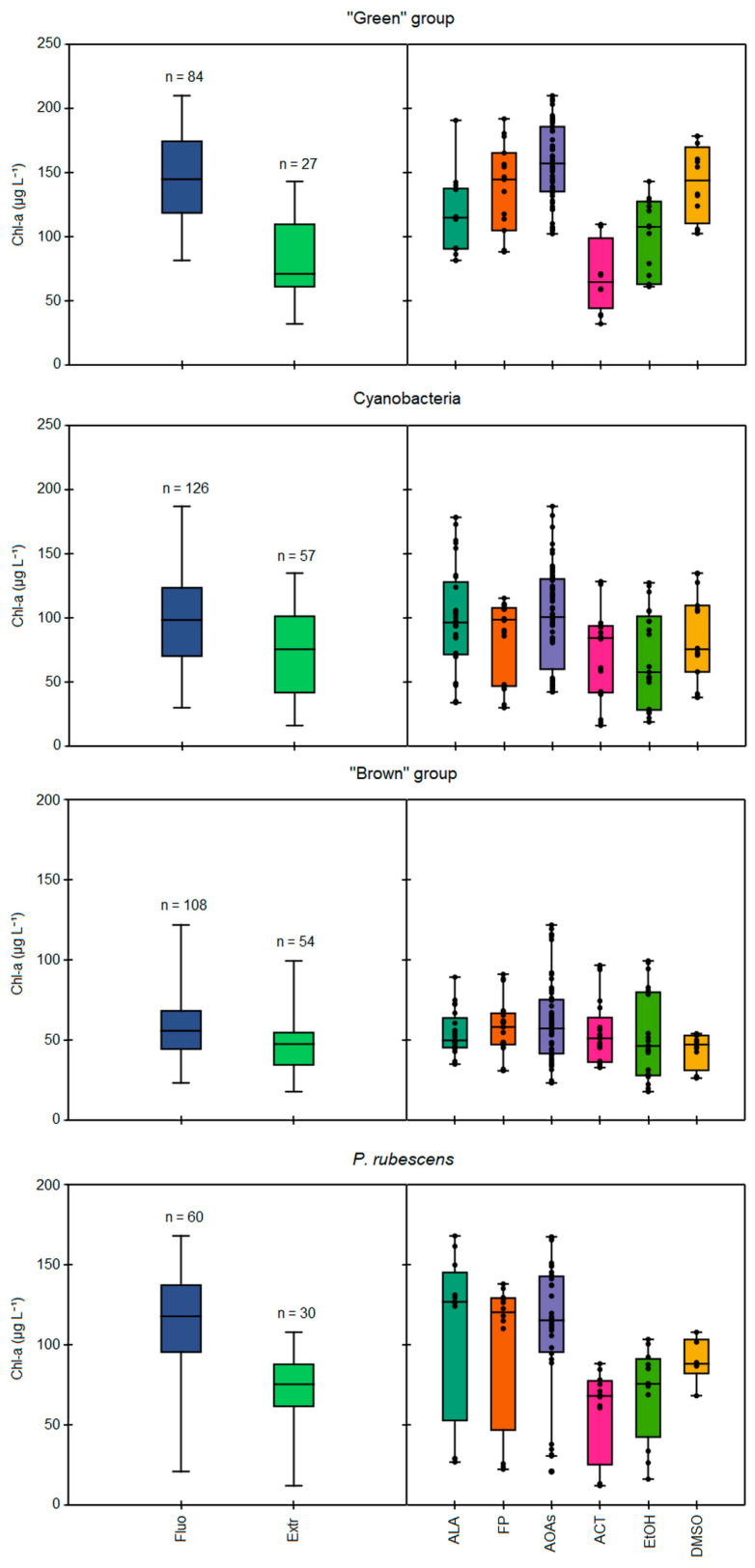
Comparison of total Chl-*a* concentrations (µg L^−1^) during the 23 simultaneous Chl-*a* detection tests, as measured by *in vivo* fluorescence and through extraction methods (on the left), or specifically according to the different models of spectrofluorometers and extraction methods (on the right), based on the target algal group, i.e., “green” group, Cyanobacteria, “brown” group, and *P. rubescens*. Fluo = all measurements performed by *in vivo* fluorescence, Extr = all measurements of extraction methods, ALA = AlgaeLabAnalyser, FP = FluoroProbe, AOAs = four models of AlgaeOnlineAnalyser, ACT = extraction with 90% acetone, EtOH = extraction with 90% ethanol, DMSO = extraction with dimethyl sulfoxide.

The comparison of total Chl-*a* concentrations measured in the monospecific cultures of each algal group by different spectrofluorometers (ALA, FP, AOAs) and after the chemical extractions with 90% acetone (ACT), 90% ethanol (EtOH), and dimethyl sulfoxide (DMSO) is reported in Figure 1. In general, higher values of Chl-*a* were obtained when using the fluorescence-based approach than the extractions (Figure 1, left side), as also reflected by the calculated ratios of the mean Chl-*a* concentrations between *in vivo* fluorescence and extraction determinations, which resulted in values above 1.0 for each scenario considered (Table 2). Significant differences among the two approaches were observed (Kruskal–Wallis, *p* < 0.05) based on the solvent used and the algal group considered (Figure 1, right side). Lower Chl-*a* concentrations in respect to the *in vivo* fluorescence determinations were obtained for the “green” group and Cyanobacteria when using ACT and EtOH (Mann–Whitney pairwise, *p* < 0.05), while extraction with DMSO gave the closest Chl-*a* concentrations to those measured with the probes, resulting in the lowest observed ratios (1.07–1.20). As for the “green” group, the extraction with ACT led to a two-fold lower mean Chl-*a* concentration than the probes (68 vs. 146 µg L^−1^), thus giving the highest ratio of 2.04 between the two approaches (Table 2). Interestingly, among the tested strains of the “green” group, the highest discrepancy among the spectrofluorometers and extractions was observed for E. gracilis regardless of the solvent used (82 vs. 162 µg L^−1^, see Figure S1). Extractions with EtOH led to generally high, but constant ratios (1.42–1.54) per algal group considered, corresponding to intermediate Chl-*a* concentrations between ACT and DMSO. Conversely, consistent Chl-*a* concentrations for the “brown” group were obtained when comparing the two approaches (Mann–Whitney pairwise, *p* > 0.05).

### 3.2. Comparison between In Vivo Fluorescence and Extraction Approaches

When comparing the different spectrofluorometer models (ALA, FP, and AOAs, see Figure 1 right side), the four AOAs generally gave higher Chl-*a* values than those obtained through extractions and with the other probe models, specifically for the “green” group and Cyanobacteria, as attested by the significant differences observed for AOA determinations with respect to ALA (Mann–Whitney pairwise, *p* < 0.05). Consequently, the Chl-*a* concentrations measured with ALA were closer to those from extractions, especially for Cyanobacteria, as also suggested by the lowest calculated ratio between the two methodologies (1.13, see Table 2). The portable sensor FP gave somewhat intermediate values compared to those measured by other probes; indeed, the calculated ratios for each algal group were in the range 1.22–1.44, resulting in values higher than ALA (1.13–1.27), but lower than AOAs (1.27–1.61). Finally, no differences among the three probe models were observed for the “brown” group. It is worth mentioning that the variability of the results obtained with the four models of AOAs was tested, resulting in non-significant differences among them for each algal group considered, except for the “brown” group (Kruskal–Wallis, *p* < 0.05).

### 3.3. Algal Group Assignment and Performance of Spectrofluorometers

**Figure 2:**
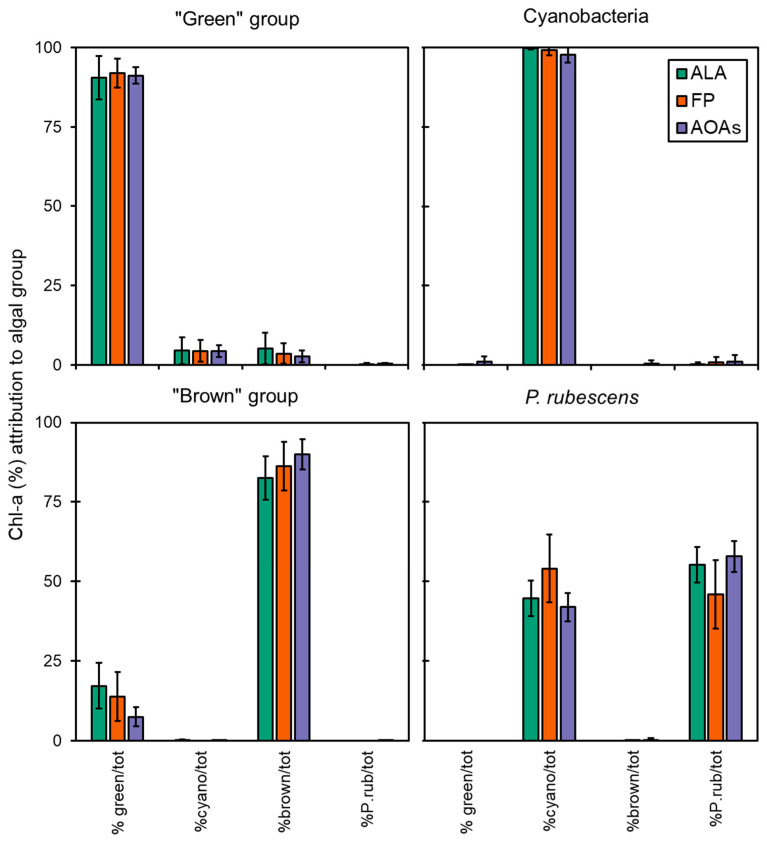
Chl-*a* concentration measured by the multi-wavelength spectrofluorometers (ALA, FP, and the four AOAs) on monoalgal cultures during intercalibration tests. Data reported are expressed as mean of the attribution percentage to four target algal groups with respect to the total Chl-*a* detected. %green/tot = percentage of Chl-*a* attributed to the “green” group; %cyano/tot = percentage of Chl-*a* attributed to Cyanobacteria; %brown/tot = percentage of Chl-*a* attributed to the “brown” group; %P.rub/tot = percentage of Chl-*a* attributed to *P. rubescens*.

The data obtained from the application of the spectrofluorometers to monospecific algal cultures of different taxa were used to determine the percentage attribution of Chl-*a* to four target algal groups (i.e., “green” group, Cyanobacteria, “brown” group, and *P. rubescens*) as shown in Figure 2. In most of the cases, almost the entire fluorescence signal of total Chl-*a* was correctly assigned to the reference algal group by the spectrofluorometers.

**Figure 3:**
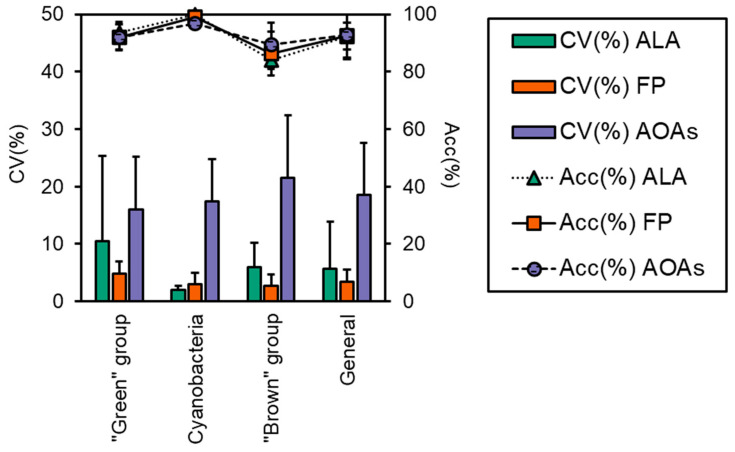
Performance evaluation of the three models of the probes (i.e., ALA, FP, and AOAs) in terms of coefficient of variation between probes’ determination of Chl-*a* and the means of all of the extraction methods (CV%) and accuracy in targeting the correct algal group (Acc%). Both CV(%) and Acc(%) were calculated for each algal group: “green” group, Cyanobacteria, and “brown” group, and for all samples, i.e., “General”.

The accuracy of each probe in targeting the correct algal group is reported in Figure 3, which was above 83% for all samples, although some minor misattributions were observed, as also shown by the calculated percent errors (Er%, Table 3). FP generally performed better in terms of variation, as attested by the CV% that was 3.4 ± 2.1 for FP and higher for the ALA and AOAs (5.7 ± 8.2 and 18.6 ± 9.0, respectively, Figure 3). As for the “green” group, more than 90% of the total Chl-*a* was correctly attributed to the target algal taxa (Figure 2), with minor misclassifications that were somehow consistent irrespective of the probe employed (6–8%, Table 3) and equally split into the Cyanobacteria and the “brown” group. For this algal group, the major variation (Figure 3) observed for ALA and AOAs was mainly due to the different Chl-*a* concentrations of one species (i.e., *E. gracilis*). The detection of Cyanobacteria monospecific cultures scored nearly perfectly with almost 100% attribution, especially with ALA and FP, hence achieving the lowest observed percent error (0–3%). Furthermore, the variation between ALA and FP with respect to the extraction methods were the lowest observed (Figure 3, 1.9% and 3.0%, respectively). Similarly, AT, the portable sensor able to only discriminate Cyanobacteria from the total Chl-*a*, correctly assigned a total of five monospecific cyanobacterial cultures, with a high percentage attribution over 98%. On the other hand, the results obtained for the “brown” group were more variable in terms of the percentage of attribution (83–90%), with a greater misclassification observed for ALA and FP that resulted in a higher percent error than the other taxa (10–16%). In this case, the portion of Chl-*a* misclassified was mainly assigned to the “green” group (8–18%). Although the accuracy of ALA and FP in targeting the “brown” group was slightly lower than the AOAs, their general performance was in line with that observed for the other algal groups (CV% 3–6%), whereas for the AOAs, this accounted for more than 20% (Figure 3).

#### Specific Detection of *P. rubescens* by Probes

All of the probes used except for AT were specifically designed by the manufacturer for the detection of *P. rubescens*. Thus, assays on the monospecific cultures of the PE-rich cyanobacterium were performed. Consistently higher concentrations of Chl-*a* than those measured through extraction determinations were obtained using all of the spectrofluorometers (Kruskal–Wallis, *p* < 0.05), and a trend similar to the other algal taxa was observed when comparing the three extraction solvents, i.e., Fluo > DMSO > EtOH > ACT (Figure 1). This was also suggested by the relatively high calculated ratios above 1.42 and, similarly, for the “green” group, the highest ratio was obtained when extracting Chl-*a* with ACT (Table 2). On the contrary, no differences among ALA, FP, and the four models of AOAs were observed for *P. rubescens* (Mann–Whitney pairwise, *p* > 0.05, see Figure 1). From a qualitative point of view, the total Chl-*a* fluorescence was almost equally divided between *P. rubescens* and general Cyanobacteria detection, resulting in attribution percentages in the range of 46–58% and 42–54%, respectively (Figure 2). Consequently, the corresponding percent error for *P. rubescens* detection with the probes was the highest observed (Table 3). Nonetheless, it is important to specify that these tests were performed by activating the probes on both the channel for the general detection of Cyanobacteria and the one dedicated to *P. rubescens*; hence, Chl-*a* was portioned among the two due to the overlapping of the emission spectra. As expected, when the channel for Cyanobacteria was turned off, the algal group assignment to *P. rubescens* performed the best, with a 100% Chl-*a* percentage attribution.

### 3.4. Comparison among Methodologies Using Freshwater Sample

**Figure 4:**
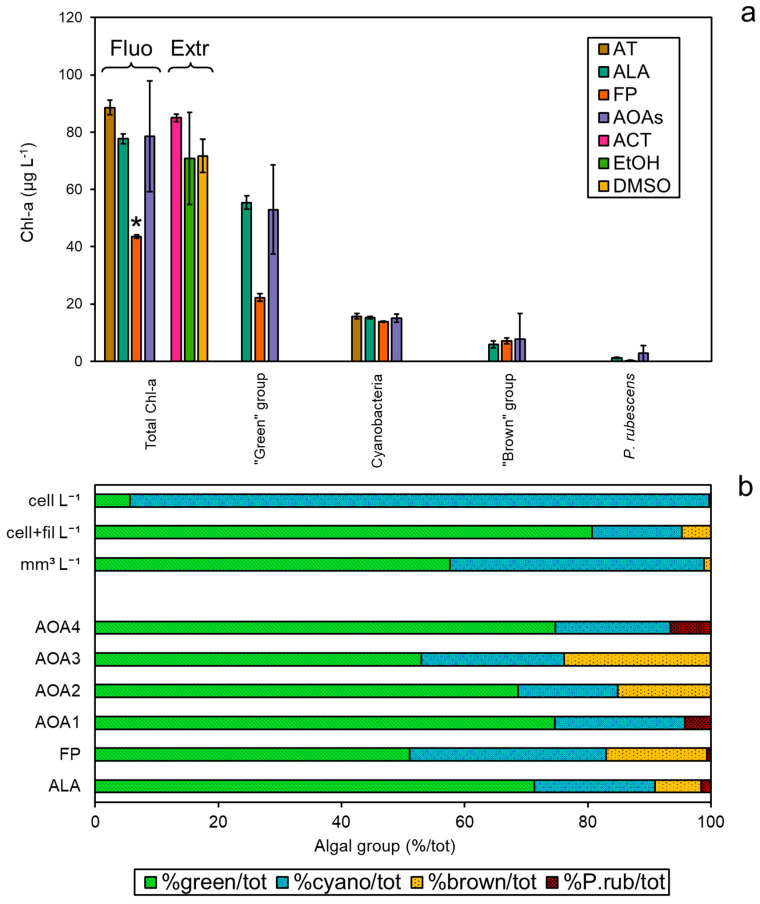
Application of the probes to a field sample collected from Reno River (Emilia-Romagna, Italy). (**a**) Total Chl-*a* concentrations (µg L^−1^) based on spectrofluorometers and extraction determinations per algal group, i.e., “green” group, Cyanobacteria, “brown” group, and *P. rubescens*. (**b**) Relative distribution of the algal groups observed per means of different methods expressed as percentage of the total, from the top to the bottom: biovolume (mm^3^ L^−1^), number of cells per liter (cell L^−1^), cells and filaments per liter (cell+fil L^−1^), and percentage of algal group attribution by the probes. * Significant comparisons for total Chl-*a* (ANOVA, *p* < 0.05).

The spectrofluorometers (AT, ALA, FP, and the four models of AOAs, AOA1–4) were simultaneously tested on a field sample collected from Reno River (Emilia-Romagna, Italy), and compared to the Chl-*a* extractions (Figure 4a). The concentrations of total Chl-*a* were found to be different between the two approaches (ANOVA, *p* < 0.05), especially after measurements with FP, which gave the lowest values against all of the other methods (Tukey’s pairwise, *p* < 0.05). As observed in the monospecific culture experiments, the Chl-*a* concentrations obtained with ALA were more in agreement with the three extraction methods (Tukey’s pairwise, *p* > 0.05). As for the attribution to the target algal taxa, similar Chl-*a* amounts assigned to Cyanobacteria (12–15 µg L^−1^) and the “brown” group (6–15 µg L^−1^) were found for each probe, corresponding to 16–23% and 8–24% of the total Chl-*a*, respectively (Figure 4b). *P. rubescens* was detected by all probes as a minor fraction to the total Chl-*a* (<7%). Interestingly, the major discrepancy was observed for the “green” group that represented the main proportion of the total Chl-*a* detected alongside Cyanobacteria. In particular, the “green” group attribution by FP was consistently lower than for the other spectrofluorometers, suggesting a possible underestimation. Phytoplankton composition expressed as a percentage of the total cell counts of the target algal groups, i.e., the “green” group, Cyanobacteria, the “brown” group, and *P. rubescens*, only partially reflected the trend observed by the spectrofluorometers (Figure 4b), as a higher number of cyanobacterial cells and a lower relative count of “green” algae were obtained compared to the *in vivo* fluorescence data (see Table S2). A Student’s *t*-test confirmed these observations, as comparisons among the number of cells (cell L^−1^) and fluorometers’ output resulted in significant differences for the “green” group, with the exception of FP, and for all probes’ output of Cyanobacteria (*p* < 0.001, Table S3). However, when filamentous cyanobacteria were counted in terms of filaments per liter (cell+fil L^−1^) instead of cells per liter (cell L^−1^), the relative abundance of algal taxa was more in agreement with the probes’ classification. Indeed, most of the comparisons between the microscopic measurements (cell+fil L^−1^) and probe outputs were not significantly different for Cyanobacteria (Table S3). Similarly, based on biovolume measurements, the “green” group accounted for 58% of the total, which mainly corresponded to *Chlamydomonas* sp. (2.994 mm^3^ L^−1^), followed by Cyanobacteria (41%), for which the main constituent was cf. *Planktothrix* sp. (1.175 mm^3^ L^−1^) (Table S2). No filaments of *P. rubescens* were observed in the sample, although other PE-containing cyanobacteria were counted, suggesting that a possible minor misattribution by the probes with respect to the “red” group could not be excluded.

## 4. Discussion

Discrepancies in Chl-*a* concentrations measured on monoalgal cultures or phytoplankton assemblages with various approaches have been previously reported and linked to specific characteristics of the chosen methodology and the sample [5,11,16,20,21,22,37]. Here, markedly lower Chl-*a* concentrations were observed when using ACT compared to the other methods, especially when cultures of “green” species were extracted, even though the number of tested strains was lower compared to the other algal groups (i.e., Cyanobacteria and the “brown” group). It is likely that a complete Chl-*a* extraction was not achieved when using ACT, as also suggested observing the filters that remained green-colored. Conversely, the poor extraction efficiency of ACT towards Chlorophyceae has been reported by other authors [38,39,40]. Sartory and Grobbelaar [41] found that alcoholic solvents (ethanol and methanol) were more suitable extractants than ACT on both Chlorophyceae and Cyanobacteria. A similar trend was also observed by Wasmund et al. [42], who reported higher extraction efficiencies of ethanol than acetone on cultures of the cyanobacterium *M. aeruginosa* and on natural phytoplankton assemblages. Although less frequently used, DMSO can be an effective Chl-*a* extractant, especially from Chlorophyceae and Cyanobacteria, as higher concentrations, which were also closer to the probes’ results, were obtained in this study with this solvent. Similar evidence was previously reported for different strains of green algae [39] and biological soil crust dominated by Cyanobacteria [43], whereas DMSO appeared to be the worst extractant compared to other solvents when used for cyanobacterial cultures of *Anabaena* sp. PCC 7120 and *M. aeruginosa* 905 [44]. Nevertheless, DMSO was found to be an optimal alternative to extract Chl-*a* from recalcitrant algae, such as Chlorophyta that are characterized by a complex multi-layered cell wall [45] that may reduce the efficiency of the extraction of various compounds, including pigments [40]. Thus, the findings of this intercalibration study confirm the high potential of DMSO as a solvent for rapid Chl-*a* determination. 

All Chl-*a* measurements performed with the spectrofluorometers (i.e., ALA, FP, and AOA) were found to be higher compared to the data obtained with extractions. Similarly, consistent overestimations of Chl-*a* concentrations were observed when using AOA on laboratory cultures and field tests, resulting in two-fold higher values than extractions with ACT, although followed by HPLC determination [46,47]. On the contrary, consistently three-times lower underestimation of Chl-*a* by FP than extractions with EtOH has been observed in tropical reservoirs, especially in Chroococcales-dominant samples [22]. A similar trend was reported by Gregor and Maršálek [5], who found slight underestimations of Chl-*a* by FP with respect to pigment extraction with EtOH in samples with Cyanobacteria dominance, resulting in a final ratio probes-to-extraction of 0.83. The same authors later reported similar lower values from FP measurements for other natural assemblages, regardless of the algal group dominance [12]. Results obtained with FP seem variable, as a more neutral ratio of 1.03 between FP and extraction determinations with ACT were found [13], whereas both higher and lower Chl-*a* concentrations when using FP were reported compared to ACT extraction [20]. The results obtained here with ALA were more in agreement with extractions, especially for Cyanobacteria. The laboratory-based nature of ALA makes it suitable for different needs with respect to FP or AOAs, which are portable and able to perform live and *in situ* measurements. Due to these characteristics and based on the results obtained here (i.e., higher accuracy and lower variation), more accurate data with ALA than with the other probes could be expected, as after sampling, this tool can be run under controlled laboratory conditions. As for ALA, few data are reported in the literature, and most of them are related to its use in research experiments to determine Chl-*a* content and photosynthetic efficiency, rather than for a comparison among detection methodologies [48,49,50,51,52], whereas a strong correlation between cyanobacterial biomass and ALA Chl-*a* was found in a Swedish lake [53]. Nonetheless, a general inconsistency between Chl-*a* quantification methodologies has been previously discussed [54], and discrepancies among *in vivo* fluorescence and extraction procedures have been related to the calibration of the bbe Moldaenke probes that are based on HPLC pigment analysis, by which the separation onto chromatograms of distinct Chl-*a* allomers occurs, resulting in an apparent lower Chl-*a* content [5]. Here, higher Chl-*a* concentrations than extraction procedures were observed, which, on the contrary, showed high variability depending on the solvent used, thus suggesting poor extraction efficiency. Since the calibration of the probes is based on the spectral “fingerprints” of representative species, a possible variation of intracellular Chl-*a* content and taxa-specific accessory pigments in our cultured microalgae and Cyanobacteria than the ones used during the manufacturer’s calibration of the probes could not be excluded. Among the tested Cyanobacteria, several strains were filamentous and had the tendency to form dense aggregates (see Figure S2); the use of *in vivo* fluorescence to detect Chl-*a* in organisms with such morphology may lead to inaccuracies in both quantification and target algal group attribution [20,23]. Among the “green” group species tested, the major discrepancy in Chl-*a* concentrations between spectrofluorometers and extraction measurements was observed for *E. gracilis*. A possible explanation for this trend is likely due to the peculiar accessory pigment content of Euglenophyceae, that can strongly vary based on growth conditions and may impact Chl-*a* emission fluorescence by increasing or decreasing it [11]. Interestingly, a similar result was previously obtained by Nguyen et al. [18], who found major deviations among AOA measurements and extraction with ACT when Chl-*a* concentrations exceeded 40 µg L^−1^, which was the case of the present study. Additionally, *E. gracilis* was the only motile organism tested during the intercalibration procedures; thus, a higher variability of light incidence on single cells than non-motile algae is expected and could subsequently be reflected in its fluorescence. 

A good assignment to the target algal group by the probes in monospecific cultures or water samples dominated by a specific organism has been generally found [7,11,12,20,46,55], although minor misattributions were observed, possibly depending on the phytoplankton community composition, probe model, or external interferences [16,37,56]. Algal group misattributions were likely due to the differences in the spectral “fingerprint” between the tested species and the manufacturer’s ones [37,57], as well as to the partial overlapping of the emission spectra of Chl-*a* and taxa-specific accessory pigments, especially PC [23]. Even though the models used were different, they shared the same detection principle, thus possibly explaining the consistency in the percent errors observed. Similarly, Escoffier et al. [37] reported comparable values of misclassifications when using an FP on monospecific algal cultures with factory-based settings (“green” group 7.8%, Cyanobacteria 0.6%, and “brown” group 15.6%). In the specific case of the “brown” group, the similar results obtained here suggest a possible underestimation of diatom biomass, which is among the major factors responsible for filter-clogging issues in drinking water treatment plants [58]. Although toxic diatoms are generally only reported in marine environments, more recently, the production of β-methylamino-L-alanine (BMAA) and its isomers, i.e., the neurotoxic non-proteinogenic amino acids produced by Cyanobacteria, has been confirmed for some cultured freshwater diatoms (e.g., *Cyclotella*, *Navicula*, and *Tabellaria*), likely as a response to environmental stress such as nitrogen starvation [59]. Due to their well-established potential toxicity, most of the studies on the improvement of multi-wavelength spectrofluorometers are focused on Cyanobacteria. Nevertheless, this study highlights the need to improve the detection of other major algal groups that are sometimes found to be problematic, adopting a precautionary approach in water management for emerging potential health risks. This is especially true considering the growing need for fast and easy methods for the monitoring of these organisms suitable for non-insiders (i.e., non-phycologists or untrained employers of drinking water companies or freshwater bodies for recreational activities). 

As stated before, the misattributions of the probes in assessing the algal groups observed here were consistent; thus, it could be possible to adjust the raw data based on this systematic error by calculating the correction factors [11,23,56]. This strategy should be employed by recalibrating the probes with species isolated from local reservoirs to obtain accurate spectral curves (i.e., the intracellular pigment ratio may differ among strains). Interestingly, misclassification rates up to 67.8% were reported when analyzing cultures of *Limnothrix redekey*, a PE-rich cyanobacterium, showing that the Chl-*a* was almost equally attributed to the “red” group and Cyanobacteria [37]. Similar results were obtained here for *P. rubescens* detection, using the specific calibration by the manufacturer. The overlapping of emission spectra was likely the cause of this misattribution; thus, if the presence of *P. rubescens* is suspected, the analysis with a specifically calibrated device should be performed by switching off the dedicated channel for Cyanobacteria and Cryptophyta. Moreover, *P. rubescens* probe-based detection can be further optimized by calibrating the devices with locally isolated species, as reported for a French lake [13].

With regard to applications using field samples, the under- or overestimation of Chl-*a* by spectrofluorometers, with respect to extractions, have been previously reported and are related to several interference sources (for a detailed list, see Bertone et al. [16]). Here, the main difference was found for the “green” group as measured by FP, which was drastically underestimated compared to the other probes. It has been generally found that the presence of algae belonging to the “green” group can hamper the detection of other target algal groups, particularly Cyanobacteria [16,18,23,56]. Based on biovolume measurements, the sample analyzed here mainly consisted of “green” algae (58%) and Cyanobacteria (41%), whereas the *in vivo* fluorescence analysis also underlined the presence of other algal taxa, i.e., from the “brown” group. Inconsistencies among taxon-specific Chl-*a* and biovolume were previously found [55,60,61], whereas other authors reported that these values were well correlated, especially for Cyanobacteria [7,11,22,62]. Although biovolumes can give detailed information on the taxa present in a sample, their calculation can be time consuming and the values obtained can be variable among the same algal group due to different cellular sizes, i.e., within the “green” group and Cyanobacteria (see Figure S2). However, this study confirmed that counting is not always the best method for expressing the relative amount of potentially toxic cyanobacteria, as they can be dramatically overestimated with respect to the other algal groups. A possible alternative is to count filamentous species as number of filaments instead of number of cells, although other limitations could not be excluded as well, for instance, the presence of irregular cyanobacterial agglomerates or heavy variation in filament lengths. Such findings may indicate that the application of generalized conversion factors allowing the quantification from Chl-*a* to algal cell enumeration should be discouraged, keeping in mind that the original calibration may not be based on the same strains as those present in local reservoirs. To the best of our knowledge, this is the first study that includes data originating from intercalibration procedures among different laboratories comparing Chl-*a* extraction using solvents and multi-wavelength spectrofluorometers. In more traditional Chl-*a* calibration circuits, dried microalgae pellets resuspended in distilled water are employed; however, this approach is not suitable for *in vivo* fluorescence. On the contrary, the use of fresh cultures could cause a certain qualitative variability due to transport and storage considering the geographic distance among the laboratories. Here, the use of monoalgal cultures in Chl-*a* intercalibration procedures has been revealed to be a valid alternative for the drinking water companies that use such fluorometers, as the differences observed were more related to the different fluorometer models rather than to the laboratories. Conversely, the calibration of the fluorometers employed in this study is performed by the manufacturer using monoalgal cultures extracted with ethanol, followed by HPLC determination of pigments in the algal extract. The results of the present study evidence that the extraction efficiency varies among the different algal groups based on the solvent. Consequently, the estimation of the Chl-*a* by probes could be affected by the general calibration of the fluorometer based on a single solvent (i.e., ethanol) and not optimized for each specific algal group. Finally, interlaboratory calibration processes are essential to ensure the correct functioning of the probes, especially those used for continuous monitoring, i.e., AOA, since technical issues or human errors could potentially be underestimated or even missed.

## 5. Conclusions

The reliability and accuracy of different multi-wavelength spectrofluorometers compared to extraction methods for Chl-*a* determination were assessed in this study. Based on the results, the following conclusions can be drawn:

- Discrepancies in Chl-*a* determination among the two approaches were observed; however, the use of strong solvents, i.e., dimethyl sulfoxide, can improve pigment extraction, and the choice of probe model can lead to more accurate results.
- The correct algal group assignment by the spectrofluorometers was achieved, especially for Cyanobacteria, suggesting their use as reliable supportive tools in drinking water monitoring.
- The misattributions observed were low and consistent; thus, the recalibration of the probes with fresh cultures of local algal and cyanobacterial strains should be encouraged to optimize their detection.
- The intercalibration approach applied here was revealed to be useful for improving the use and performance of *in vivo* fluorescence tools for the monitoring of freshwater phytoplankton and Cyanobacteria, and to gain a better understanding of their correct functioning by non-trained staff, ultimately ensuring an improvement of drinking water management aimed at preventing environmental and public health issues related to the presence of harmful cyanobacteria.

## Figures and Tables

**Table 1:** List and sources of algae and cyanobacteria used in the present work. Suppliers: SAG = Culture Collection of Algae of Göttingen University; CCAP = Culture Collection of Algae and Protozoa; NIVA-CCA = Norwegian Institute for Water Research (NIVA) Culture Collection of Algae.

Algal Group	Strain Name	Strain Details	Supplier or Ref.
“Green” group	*Euglena gracilis*	SAG 1224-5/25	SAG
	*Chlorella vulgaris* (“C”)	CCAP 211/52	CCAP
	*Chlorella vulgaris* (“K”)	K-1801	NIVA-CCA
	*Desmodesmus communis*	Locally isolated (artificial freshwater pond, Forlì-Cesena, Italy)	[24]
Cyanobacteria	*Planktothrix rubescens*	CCAP 1459/22	CCAP
	*Planktothrix agardhii*	CCAP 1459/16	CCAP
	*Microcystis aeruginosa*	CCAP 1450/10	CCAP
	cf. *Dolichospermum* sp.	Locally isolated (Reno River, Ravenna, Italy)	This study
	cf. *Anabaena* sp.	Locally isolated (Reno River, Ravenna, Italy)	This study
“Brown” group	*Stephanodiscus hantzschii*	CCAP 1079/4	CCAP
	*Fragilaria crotonensis*	CCAP 1029/20	CCAP
	cf. *Cyclotella* sp.	Locally isolated (Reno River, Ravenna, Italy)	This study

**Table 2:** Ratios of mean Chl-*a* concentrations (µg L^−1^) measured by spectrofluorometers and extraction determinations for each target algal group. Fluo = mean of *in vivo* fluorescence determinations; Extr = mean of extracts. Determinations performed with the four models of AOAs are grouped together.

Algal Group	Fluo/Extr	ALA/Extr	FP/Extr	AOAs/Extr	Fluo/ACT	Fluo/EtOH	Fluo/DMSO
“Green” group	1.44 ± 0.45	1.27 ± 0.52	1.44 ± 0.42	1.61 ± 0.44	2.04 ± 0.62	1.46 ± 0.50	1.07 ± 0.34
Cyanobacteria	1.25 ± 0.19	1.13 ± 0.25	1.25 ± 0.19	1.36 ± 0.21	1.21 ± 0.16	1.54 ± 0.43	1.12 ± 0.21
“Brown” group	1.23 ± 0.27	1.20 ± 0.33	1.22 ± 0.35	1.27 ± 0.20	1.18 ± 0.16	1.53 ± 0.72	1.20 ± 0.22
*P. rubescens*	1.58 ± 0.12	1.66 ± 0.16	1.48 ± 0.17	1.58 ± 0.22	1.88 ± 0.22	1.42 ± 0.25	1.50 ± 0.17

**Table 3:** Misattribution rate of each spectrofluorometer in targeting the correct algal group on monospecific cultures, calculated as percent error (Er%). Data reported are based on all simultaneous tests performed and joined together based on the target algal group. AT provides total and cyanobacterial Chl-*a*; thus, the percent error was only reported for Cyanobacteria.

Target Algal Group	ALA (Er%)	FP (Er%)	AOAs (Er%)	AT (Er%)
“Green” group	6.4 ± 4.3	8.1 ± 4.6	8.0 ± 3.7	-
Cyanobacteria	0.2 ± 0.5	0.9 ± 1.6	3.1 ± 3.6	1.0 ± 1.4
“Brown” group	16.1 ± 7.7	13.8 ± 7.6	10.7 ± 3.1	-
*P. rubescens*	44.7 ± 5.6	54.1 ± 10.7	42.5 ± 5.2	-

## Data Availability

The data presented in this study are available on request from the corresponding authors. The data are not publicly available due to the privacy policies of the companies/laboratories that carried out the surveys.

## Supplementary Materials

The following supporting information can be downloaded at: https://www.mdpi.com/article/10.3390/ijerph192114075/s1, Figure S1: Chl-*a* concentration (µg L^−1^) obtained for each sample with the three models of spectrofluorometers (i.e., ALA, FP, AOAs) and the three solvent extractions methods (i.e., ACT, EtOH, DMSO); Figure S2 Microscopic photographs of the algae and cyanobacteria employed in the study. Table S1: List and specifications of spectrofluorometers employed in this study; Table S2: Qualitative and quantitative analysis and biovolume of phytoplankton and Cyanobacteria in the natural freshwater sample; Table S3: Values of Student’s *t*-test from two-tailed goodness-of-fit test (confidence level 99.9%, *p* < 0.001).
