# Supplementary material for: Improvement of In Vivo Fluorescence Tools for Fast Monitoring of Freshwater Phytoplankton and Potentially Harmful Cyanobacteria
Source: Int J Environ Res Public Health. 2022 Oct 28;19(21):14075. doi: 10.3390/ijerph192114075 (PMC9658348; doi:10.3390/ijerph192114075)
Supplement: Supplementary file 1 [file ijerph-19-14075-s001.zip › ijerph-1966027-supplementary.pdf]

Supplementary Materials:

Improvement of *In Vivo* Fluorescence Tools for Fast Monitoring of Freshwater Phytoplankton and Potentially Harmful Cyanobacteria

Mara Simonazzi [1,*], Laura Pezzolesi [1,2,*], Franca Guerrini [1], Silvana Vanucci [3], Giancarlo Graziani [4], Ivo Vasumini [4], Andrea Pandolfi [4], Irene Servadei [5] and Rossella Pistocchi [1,2]

[1] Department of Biological, Geological and Environmental Sciences (BiGeA), University of Bologna, Via S'Alberto 163, 48123 Ravenna, Italy
[2] Interdepartmental Centre for Industrial Research in Renewable Resources, Environment, Sea and Energy (CIRI-FRAME), University of Bologna, Via S'Alberto 163, 48123 Ravenna, Italy
[3] Department of Chemical, Biological, Pharmaceutical and Environmental Sciences (ChiBioFarAm), University of Messina, Viale Ferdinando d'Alcontres 31, 98166 Messina, Italy
[4] Romagna Acque Società delle Fonti S.p.a., Piazza Orsi Mangelli 10, 47122 Forlì, Italy
[5] Fondazione Centro Ricerche Marine, Viale A. Vespucci, 2, 47042 Cesenatico, Italy
* Correspondence: mara.simonazzi2@unibo.it (M.S.); laura.pezzolesi@unibo.it (L.P.); Tel.: +39-0544-937373 (M.S. & L.P.)

a

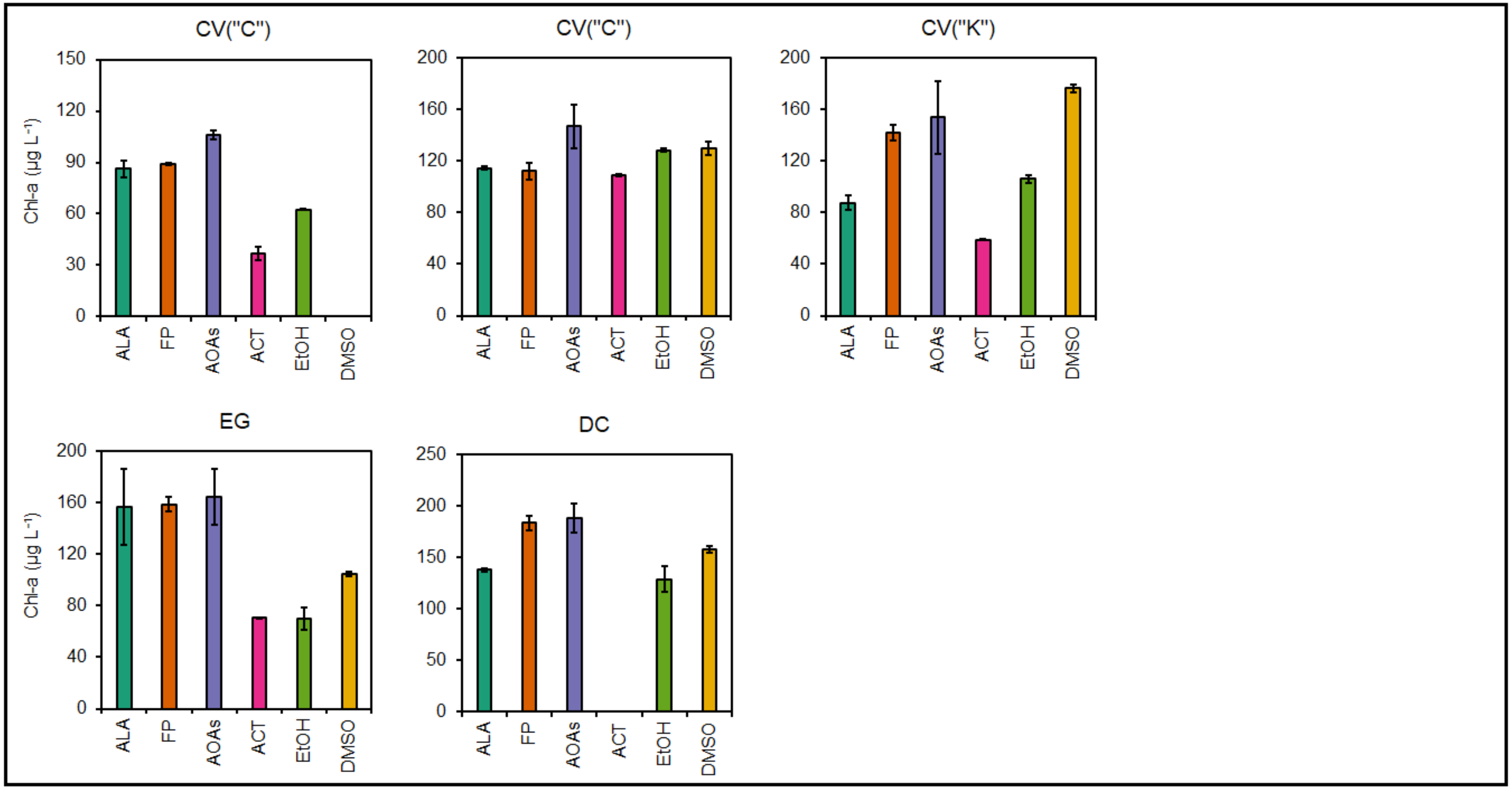
CV("C")
CV("C")
CV("K")
EG
DC
Chl-a (μg L⁻¹)
ALA
FP
AOAs
ACT
EtOH
DMSO
0
30
60
90
120
150
40
80
160
200
50
100
250

b

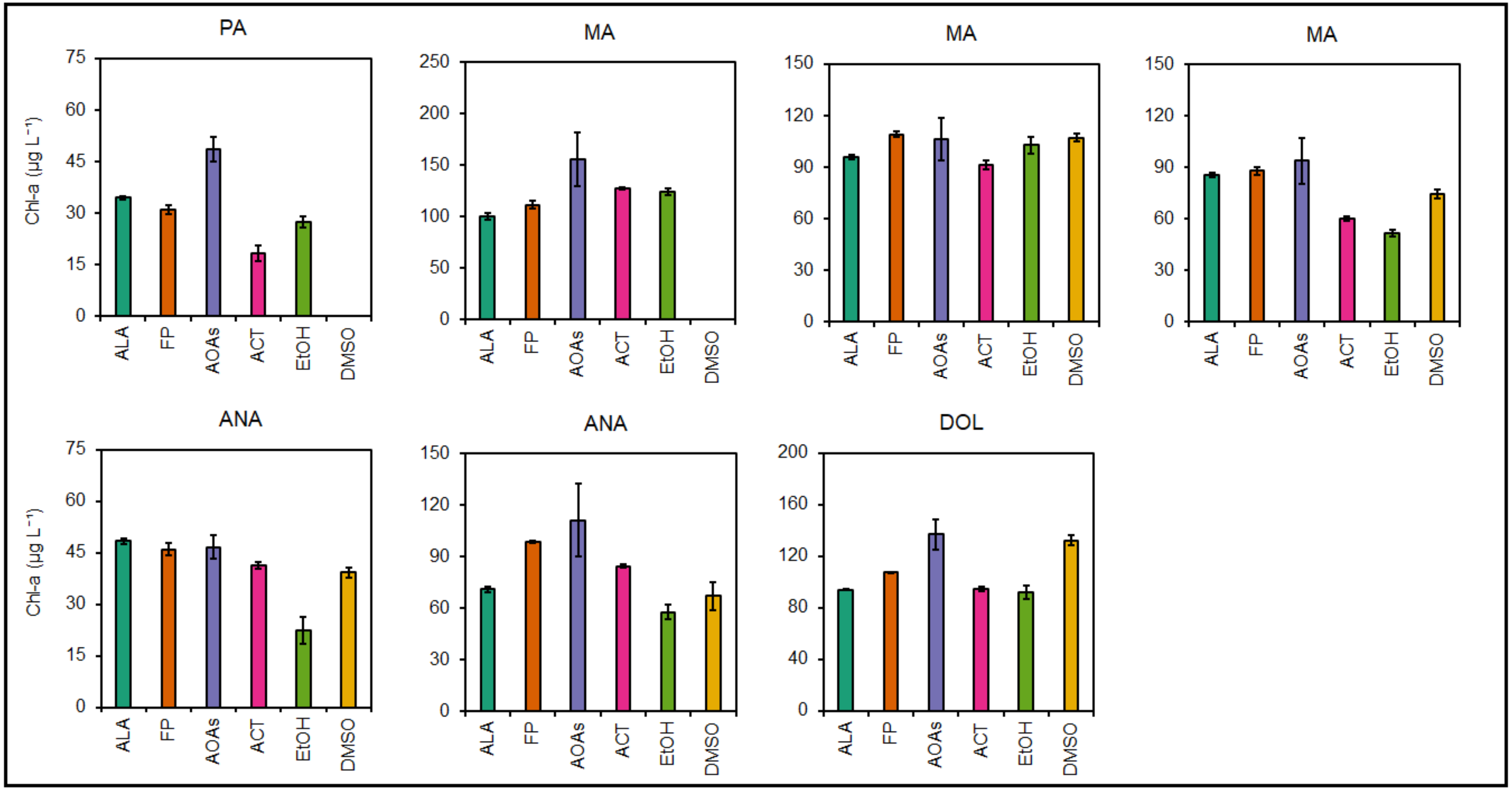

c

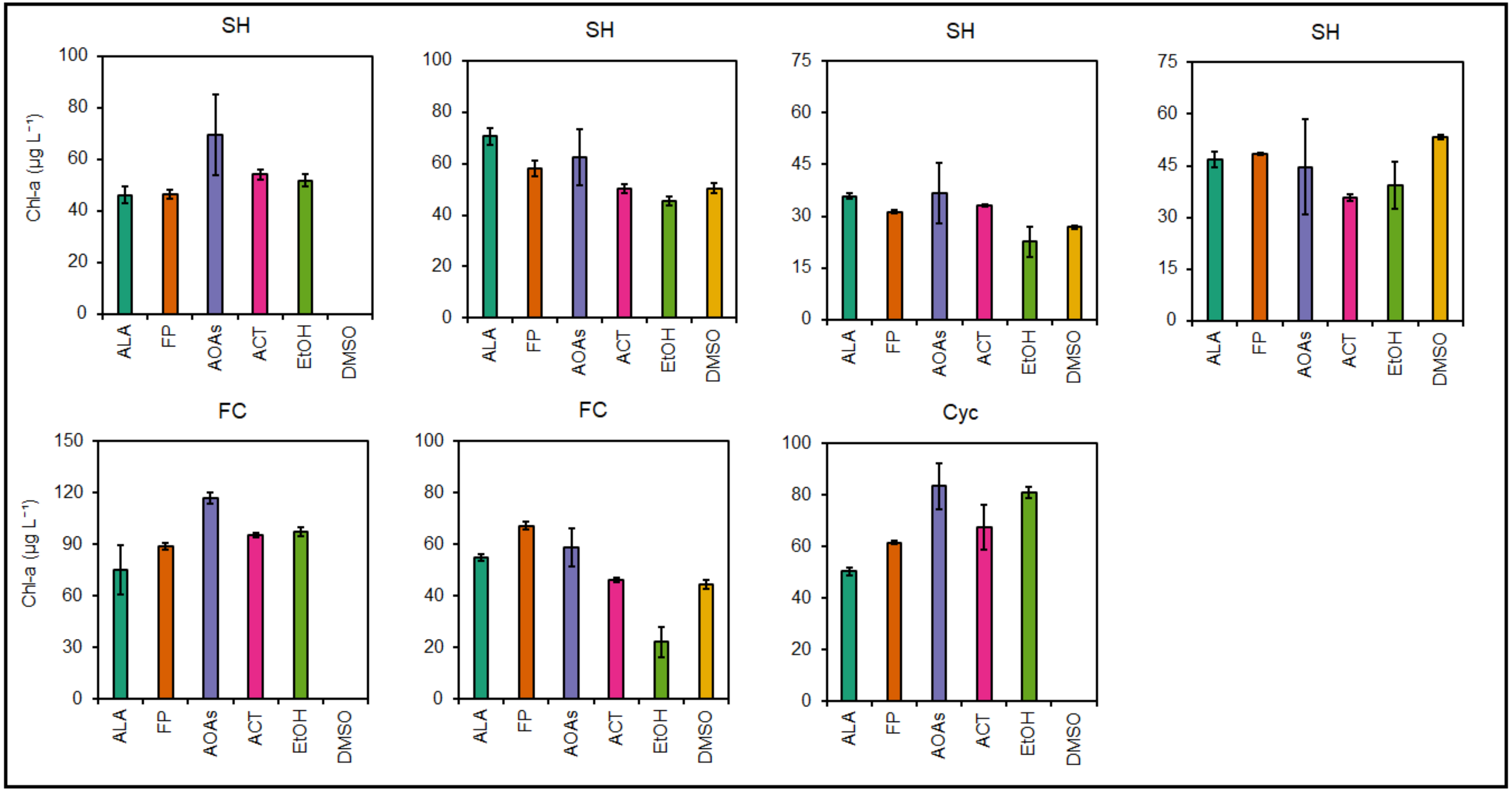
SH
SH
SH
SH
FC
FC
Cyc
Chl-a (µg L⁻¹)
Chl-a (µg L⁻¹)
ALA
FP
AOAs
ACT
EtOH
DMSO

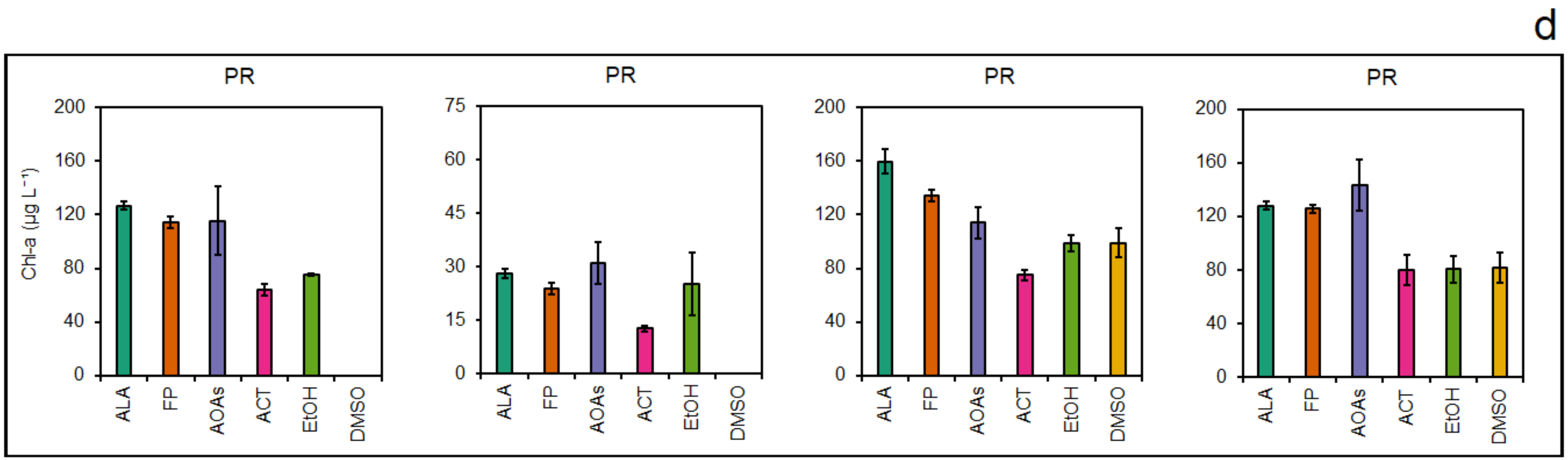

Figure S1. Chl-a concentration (µg L^{-1}) obtained for each sample with the three models of spectrofluorometers (i.e. ALA, FP, AOAs) and the three solvent extractions methods (i.e. ACT, EtOH, DMSO). (a) "Green" group, CV("C") = *Chlorella vulgaris* (CCAP strain), CV("K") = *Chlorella vulgaris* (NIVA-CCA strain), EG = *Euglena gracilis*, DC = *Desmodesmus communis*, (b) Cyanobacteria, PA = *Planktothrix agardhii*, MA = *Microcystis aeruginosa*, DOL = *Dolichospermum* sp., ANA = *Anabaena* sp., (c) "Brown" group, SH = *Stephanodiscus hantzschii*, Cyc = *Cyclotella* sp., FC = *Fragilaria crotonensis*, and (d) "Red" group, PR = *Planktothrix rubescens*.

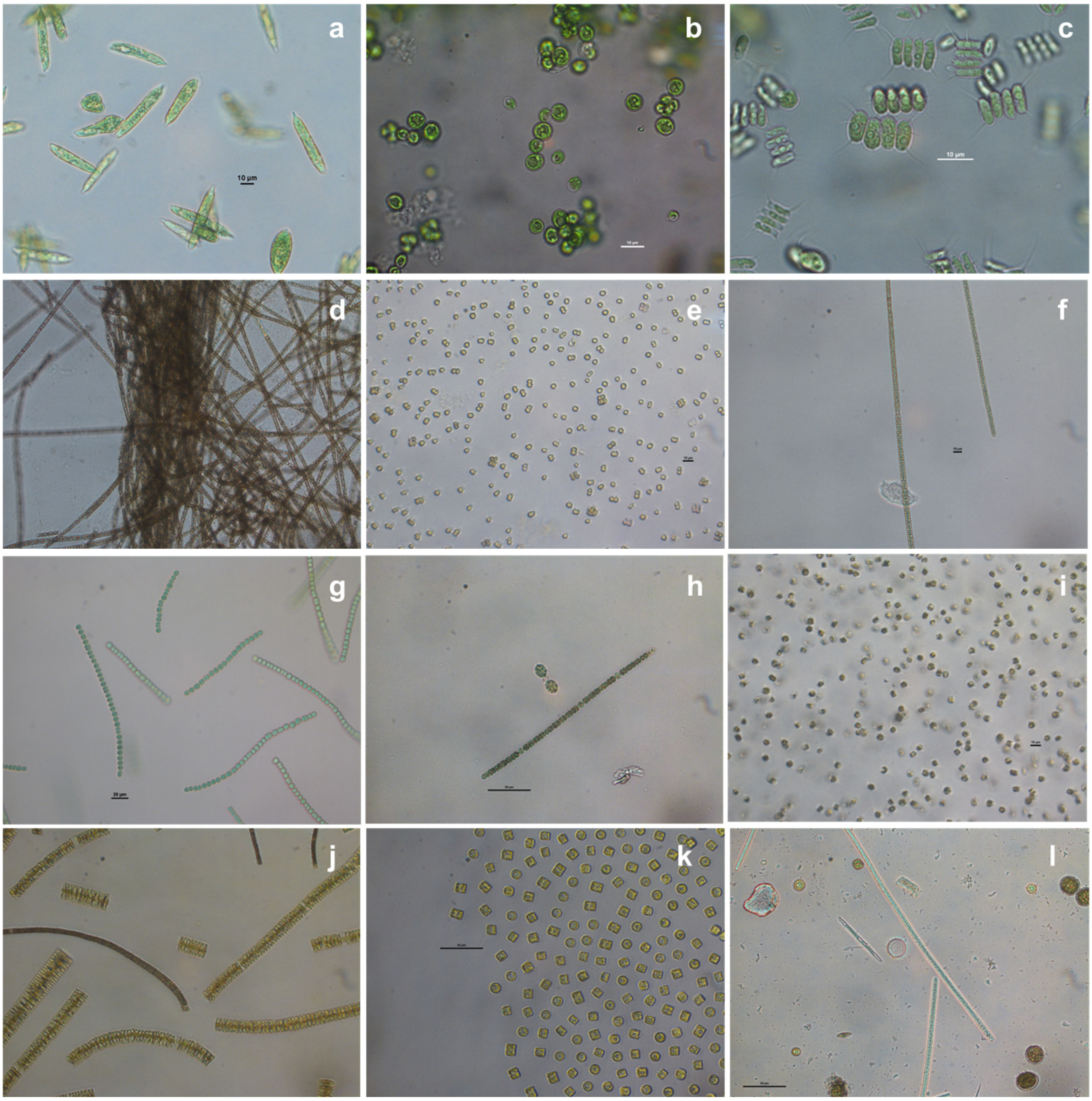

Figure S2. Microscopic photographs of the algae and cyanobacteria employed in the study. (a) *Euglena gracilis*; (b) *Chlorella vulgaris*; (c) *Desmodesmus communis*; (d) *Planktothrix rubescens*; (e) *Microcystis aeruginosa*; (f) *Planktothrix agardhii*; (g) cf. *Anabaena* sp.; (h) cf. *Dolichospermum* sp.; (i) *Stephanodiscus hantzschii*; (j) *Fragilaria crotonensis*; (k) cf. *Cyclotella* sp.; (l) natural freshwater sample from Reno river (Emilia-Romagna, Italy). All images were taken using a magnification of 320x, except for (e) and (i) (magnification 400x), and (b) and (c) (magnification 1000x).

Table S1. List and specifications of spectrofluorometers employed in this study. Lab1-5 were the distinct laboratories employing the spectrofluorometric probes. ALA = AlgaeLabAnalyser, FP = FluoroProbe, AOA = AlgaeOnlineAnalyser (4 distinct probes with the same specifications), AT = AlgaeTorch.

Laboratory	Probe	Number of LEDs	Emission (nm)	Excitation (nm)	Chl-a range (µg L^{-1})	Resolution (µg L^{-1})
Lab1	ALA	6	680	370 – 470 – 525 – 570 – 590 – 610	0 – 200	0.01
Lab2	FP	6	680	370 – 470 – 525 – 570 – 590 – 610	0 – 200	0.01
Lab2	AOA1	6	680	370 – 470 – 525 – 570 – 590 – 610	0 – 200	0.01
Lab3	AOA2	6	680	370 – 470 – 525 – 570 – 590 – 610	0 – 200	0.01
Lab4	AOA3	6	680	370 – 470 – 525 – 570 – 590 – 610	0 – 200	0.01
Lab5	AOA4	6	680	370 – 470 – 525 – 570 – 590 – 610	0 – 200	0.01
Lab1	AT	7	680	470 – 525 – 570 – 610	0 – 200	0.1

Table S2. Qualitative and quantitative analysis and biovolume of phytoplankton and Cyanobacteria in the natural freshwater sample. Total phytoplankton count is reported as cell L^{-1}, while for cyanobacterial filaments numbers are expressed as cell-per-filaments L^{-1} or filaments L^{-1}

Algal group (based on probes)	Class	Order	Family	Alga	cell L^{-1}	filaments L^{-1}	mm³ L^{-1}
"Green"	Chlorophyceae	Chlamydomonadales	Chlamydomonadaceae	*Chlamydomonas* sp.	5.72×10^6	–	2.994
	Chlorophyceae	Sphaeropleales	Scenedesmaceae	*Scenedesmus* spp.	1.25×10^5	–	0.011
	Chlorophyceae	Sphaeropleales	Scenedesmaceae	*Coelastrum* sp.	7.90×10^4	–	0.006
	Chlorophyceae	Sphaeropleales	Hydrodictyaceae	*Tetraedron* sp.	9.12×10^3	–	0.001
	Chlorophyceae	Sphaeropleales	Hydrodictyaceae	*Pediastrum* sp.	9.73×10^4	–	0.020
	Chlorophyceae	Sphaeropleales	Selenastraceae	*Monoraphidium* sp.	8.51×10^4	–	0.004
	Zygnematophyceae	Desmidiales	Desmidiaceae	*Staurastrum* sp.	6.08×10^3	–	0.005
	Trebouxiophyceae	Chlorellales	Oocystaceae	*Willea* sp.	9.73×10^4	–	0.007
	Xanthophyceae	Tribonematales	Tribonemataceae	*Tribonema* sp.	9.30×10^5	–	0.622
Cyanobacteria	Cyanophyceae	Chroococcales	Chroococcaceae	*Chroococcus* sp.	7.30×10^4	–	0.002
	Cyanophyceae	Synechococcales	Merismopediaceae	*Merismopedia* sp.	1.34×10^5	–	0.001
	Cyanophyceae	Oscillatoriales	Microcoleaceae	cf. *Planktothrix* sp.	5.04×10^7	6.60×10^5	1.175
	Cyanophyceae	Nostocales	Aphanizomenonaceae	*Dolichospermum* sp.	3.89×10^5	1.52×10^4	0.025
	Cyanophyceae	Nostocales	Aphanizomenonaceae	*Cuspidothrix* sp.	2.62×10^6	6.69×10^4	0.093
	Cyanophyceae	Nostocales	Aphanizomenonaceae	*Raphidiopsis* sp.	1.43×10^7	1.37×10^5	0.505
	Cyanophyceae	–	–	filaments (< 1 µm)	5.16×10^7	2.10×10^5	0.825
"Brown"	Mediophyceae	Stephanodiscales	Stephanodiscaceae	*Cyclotella* sp.	5.47×10^4	–	0.036
	Bacillariophyceae	Bacillariales	Bacillariaceae	*Nitzschia* spp. (< 5 µm)	3.50×10^5	–	0.006
	Dinophyceae	Peridiniales	–	Peridinales indet.	1.52×10^4	–	0.031
"Red"	Cryptophyceae	Cryptomonadales	–	Cryptomonadales indet.	1.03×10^5	–	0.019
Other	Other	–	–	Other (< 20 µm)	5.78×10^4	–	0.003
				TOTAL	1.27×10^8	9.02×10^6	6.392

Table S3. Values of Student's *t* from two-tailed goodness-of-fit test (confidence level 99.9%, $p < 0.001$). Values were calculated comparing expected microscopic measurements (i.e. cell counting, cell and filaments counting, biovolume) to observed fluorometric outputs, based on algal group assignment (percentage on total) in the field freshwater sample. Percentage of Chl-a or microscopic measurements attributed to "green" group (%green/tot), Cyanobacteria (%cyano/tot), "brown" group (%brown/tot) and *P. rubescens* (%P.rub/tot). Significant *t*-values are reported in bold.

Algal group	Microscopic measurement	ALA	FP	AOA1	AOA2	AOA3	AOA4	AT	Fluo
%green/tot	cell L^{-1}	**62.85**	31.32	**573.12**	**941.68**	**178.42**	**114.27**	n.d.	**25.03**
	cell+fil L^{-1}	-8.80	-20.35	**-48.63**	**-218.84**	**-104.08**	-9.65	n.d.	**-6.45**
	mm^3 L^{-1}	13.10	-4.56	**141.39**	**135.86**	-17.74	28.22	n.d.	3.17
%cyano/tot	cell L^{-1}	**-368.92**	**-128.10**	**-428.77**	**-939.19**	**-209.15**	**-317.92**	**-152.15**	**-57.90**
	cell+fil L^{-1}	24.02	**35.53**	**37.34**	10.85	25.18	16.67	6.10	**5.58**
	mm^3 L^{-1}	**-107.30**	-19.15	**-118.44**	**-306.66**	**-53.13**	**-95.15**	**-46.79**	**-15.64**
%brown/tot	cell L^{-1}	7.17	11.60	n.d.	**126.19**	**55.51**	n.d.	n.d.	**4.71**
	cell+fil L^{-1}	2.72	8.38	n.d.	**86.93**	**44.96**	n.d.	n.d.	2.62
	mm^3 L^{-1}	6.35	11.00	n.d.	**118.94**	**53.56**	n.d.	n.d.	**4.33**
%p.rub/tot	cell L^{-1}	10.78	1.99	**72.16**	n.d.	1.00	12.87	n.d.	3.64
	cell+fil L^{-1}	10.78	1.99	**72.16**	n.d.	1.00	12.87	n.d.	3.64
	mm^3 L^{-1}	10.78	1.99	**72.16**	n.d.	1.00	12.87	n.d.	3.64
